# A physicians' compliance in identifying patients’ as drivers and providing advice on the Driver & Vehicle Licencing Agency (DVLA) guidelines

**DOI:** 10.1192/bjo.2021.246

**Published:** 2021-06-18

**Authors:** Christiana Elisha-Aboh, Amy Seukeran, Phuong Pham, Mohammad Musabbir, Helen Turner

**Affiliations:** Leeds and York Partnership NHS Foundation Trust

## Abstract

**Aims:**

The DVLA has strict guidelines regarding how long a driver should stay off driving when they have certain mental health illnesses or severity of symptoms. It is difficult to give such advice if we are unaware of the patients’ that drive; especially when they do not volunteer this information for various reasons.

This audit was aimed at identifying people who have been admitted to the Ward 3 at the Mount Hospital and if they were asked about driving. The audit also looked at whether there were discussions around the driving requirements and DVLA guidelines in terms of their mental health diagnosis. The expected outcome of this project was to improve information gathering when clerking in a new patient and to ensure that elderly patients’ who drive are made aware of the DVLA guidelines.

**Method:**

This audit retrospectively examined the care of 50 patients on Ward 3 at the Mount Hospital, a mixed acute psychiatric ward for older people, between 1st April 2020 and 11th November 2020. All patients’ aged 65 years and over who were on admission within that period were audited. Data collection took place between 17th November and 17th December 2020; this involved reviewing patient records throughout their inpatient stay including paper notes and electronic records (on Care Director). Results were compiled using a pre-determined data collection tool and analysed using Microsoft Excel. The audit used the standards within the DVLA Guidance- Psychiatric Disorders: Assessing fitness to drive.

**Result:**

Only 1 (2%) patient had sufficiently documented evidence around driving and the impact of psychotropic medication on driving. DVLA information was given verbally in 3 (9%) patients and only 2 patients had this information passed on to their General Practitioner (GP). Only 3 (6%) patients were made aware of the DVLA guidelines and 2 (4%) patients made aware of their obligation to inform the DVLA

**Conclusion:**

Generally, the compliance of psychiatrists in identifying all patients’ who drive is poor and seems even worse with elderly patients’. There was little documented evidence that patients were asked about their driving status on or during their admission, were given verbal or written information, had discussions around the impact of medication on driving or informed about their obligation to notify the DVLA. This study provides opportunity to improve practice by educating the medical workforce and raising awareness within the wider team. There also needs to be greater involvement and communication with GPs when completing discharge summaries.

